# Twisto-Electrochemical Activity Volcanoes in Trilayer Graphene

**DOI:** 10.1021/jacs.4c03464

**Published:** 2024-06-03

**Authors:** Mohammad Babar, Ziyan Zhu, Rachel Kurchin, Efthimios Kaxiras, Venkatasubramanian Viswanathan

**Affiliations:** † Department of Mechanical Engineering, University of Michigan, Ann Arbor, Michigan 48109, United States; ‡ Stanford Institute of Materials and Energy Science, SLAC National Accelerator Laboratory, Menlo Park, California 94025, United States; § Department of Physics, Harvard University, Cambridge, Massachusetts 02138, United States; ∥ Department of Materials Science and Engineering, Carnegie Mellon University, Pittsburgh, Pennsylvania 15213, United States; ⊥ Department of Aerospace Engineering, University of Michigan, Ann Arbor, Michigan 48109, United States

## Abstract

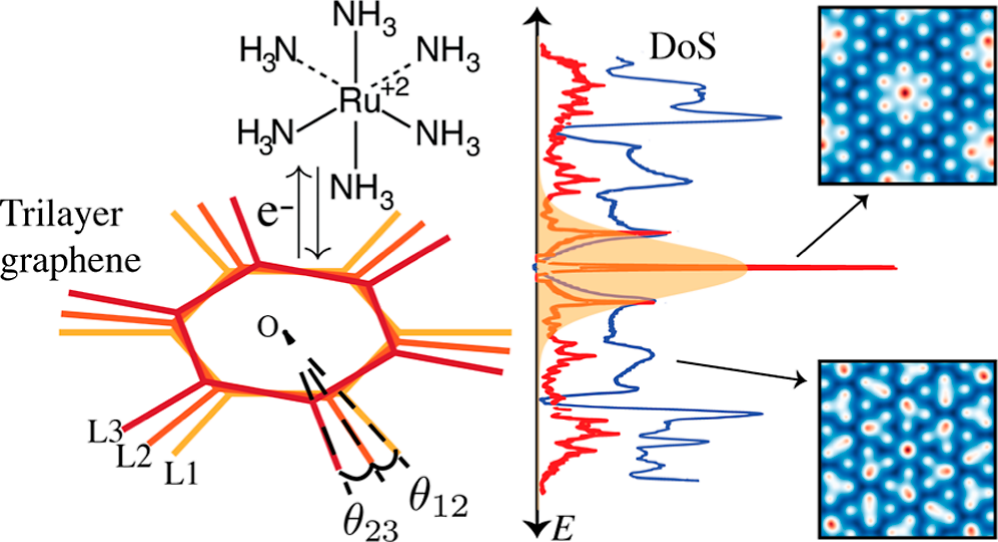

In this work, we develop a twist-dependent electrochemical activity map, combining a low-energy continuum electronic structure model with modified Marcus–Hush–Chidsey kinetics in trilayer graphene. We identify a counterintuitive rate enhancement region spanning the magic angle curve and incommensurate twists in the system geometry. We find a broad activity peak with a ruthenium hexamine redox couple in regions corresponding to both magic angles and incommensurate angles, a result qualitatively distinct from the twisted bilayer case. Flat bands and incommensurability offer new avenues for reaction rate enhancements in electrochemical transformations.

## Introduction

Enhancing electrochemical reaction rates is critical for chemical transformations,^1^ energy conversion, and energy storage.^2^ Electrochemical reactions at electrode–electrolyte interfaces are often controlled by modifying the substrate, thereby tuning the adsorption energy to reach peaks of activity “volcanoes”.^3^ Another less-studied approach to enhancing electrode–electrolyte reactions is through modifying the electronic density of states (DoS) of the electrode such that larger numbers of states coincide with the transitions of the redox couple.^4,5^ For instance, it has been shown that in single-molecule reactions with metallic electrodes (such as gold or copper), rates can increase with increasing surface DoS near the Fermi level.^6,7^

The interfacial chemical reactivity of few-layer graphene is significant for nanoscale electrochemical devices and electrocatalysis.^8−10^ Compared to other types of defects, inducing a topological twist in graphene layers introduces unique disorders and interlayer interactions giving rise to a host of exotic physical, electronic, and optical properties.^11^ These properties such as correlated insulated states,^12^ unconventional superconductivity,^13^ and orbital magnetism^14^ are primarily associated with the weakly dispersive and highly peaked “flat bands” that emerge at the magic angle (MA) ∼1.1^°^ twist in bilayer graphene. Such materials thus offer the possibility of controllable enhancement of the DoS by modifying the twist angle.^15,16^ In a recent work, we showed that twisted bilayer graphene (tBLG) can be used to control the reaction rates with a canonical redox couple, producing an activity volcano of an order of magnitude enhanced rates relative to the untwisted Bernal stacked graphene.^17^ However, the reaction rate maximum was confined close to the MA, which would present practical device fabrication challenges in ensuring precise and uniform twists in real space.

In this work, we analyze the electrochemical activity contour in twisted trilayer graphene (tTLG), which has unique electronic and mechanical relaxation properties associated with incommensurability (i.e., the induced moiré cells of the individual pairs of layers cannot be made to coincide as integer multiples)^18,19^ compared to tBLG. We use a general momentum-space model that incorporates these properties to compute the DoS as a function of twist.^18^ We then map the electron transfer rates given by the overlap of DoS with the redox couple states and electron/hole occupations under a modified Marcus–Hush–Chidsey model.^5^ Finally, we modulate the temperature and redox couple parameters to adjust enhancement and magnitude shifts in the activity volcano.

We show that the rate enhancement is not necessarily localized at the MA in multilayer 2D systems, which enables better flexibility over a range of electrochemical and redox couple parameters. In tTLG, the addition of a second twist angle creates the possibility of breaking mirror symmetry and forming incommensurate lattice patterns.^20^ We find that the effect of this incommensurability on the electronic states in the material widens the rate enhancement regime to ≥1^°^ at room temperature. We also study the effect of variations in conditions such as temperature and applied voltage as well as materials properties such as redox couple potential and reorganization energy. Incommensurate angles can be realized in twisted multilayer graphene and other 2D materials such as chalcogenides to achieve very high rate amplification.

A general scale for electrochemical reaction rates is given by the heterogeneous rate constant (*k*_0_), typically computed via the Butler–Volmer (BV) equation.^21,22^ At higher rates, the linearity assumption in BV breaks down and Marcus theory is needed, which utilizes a second-order free energy expansion.^22−26^ Chidsey introduced a further modification to account for electron and hole occupation functions, known today as the Marcus–Hush–Chidsey (MHC) model.^27,28^ The MHC model assumes a constant, energy-independent density of states. This assumption is relaxed in its extended versions^5,29^ for better predictions at high overpotentials^30^ and applications like reactivity of graphene edge states and defects^31,32^ or lithium stripping and electrodeposition.^5^

For materials with sharply varying DoS such as twisted 2D systems and others exhibiting flat bands, it is crucial to incorporate DoS effects on the interfacial kinetics with an electrolyte. Due to the large number of states at the magic angle, twisted graphene has the potential for exceptionally high rate enhancement comparable to bulk graphite.^17^ We propose that a significant enhancement of the reaction rate takes place when the redox couple energy levels line up with the energy of electrode states whose density has been increased by the tTLG. In this process, we assume an inactive substrate like the rotationally misaligned and insulating hBN bottom layer^33,34^ as used earlier^17,35^ to avoid charge-transfer doping^36^ or effect on the electrode DoS.

## Methods

**Figure 1:**
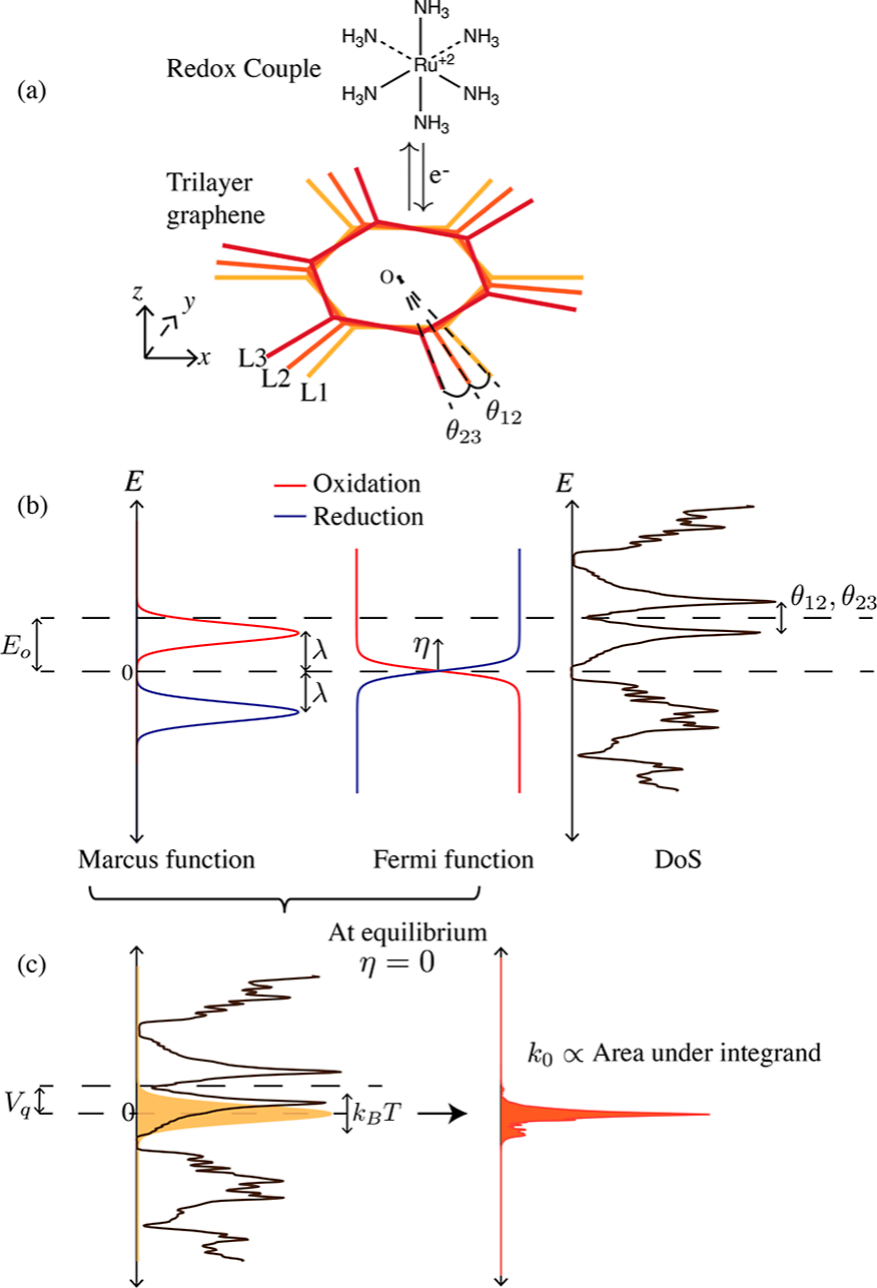
Schematic of the reaction system (a) with the trilayer graphene twisted by θ_12_, θ_23_, exchanging electron from a redox couple (RuHex) approaching from *z*. The extended Marcus–Hush–Chidsey model (b) has three factors in the integrand: the Marcus term, Fermi function, and the DoS of the electrode as shown from left to right. All tunable parameters are labeled in (b). Marcus term and the Fermi function illustrated with the same color in (b) multiply to give either oxidation (red) or reduction (blue) filters, identical at equilibrium as indicated in yellow (c). The filter overlaps with the DoS (black) shifted by quantum capacitance voltage *V*_q_. The equilibrium rate is proportional to the area under the product of the three factors.

We study the electron transfer dynamics between twisted trilayer graphene in contact with a redox couple 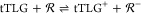
1approaching from the *z* direction, as illustrated in the schematic (Figure 1a). We use ruthenium hexamine (Ru^+3^(NH_3_)_6_, RuHex hereafter) in aqueous KCl solution as our reference redox couple whose formal potential is closest to the charge neutrality point (CNP) of multilayer graphene.^17^

The extended MHC theory^5,31^ predicts the reaction rate given the reorganization energy (λ), applied overpotential (η), temperature (*T*), and DoS  of the electrode
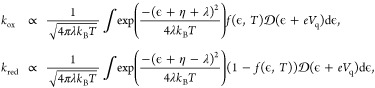
2where *f* is the Fermi–Dirac distribution. The integrand has three factors: (“Marcus-like”) redox couple states, electron or hole occupation probabilities, and the DoS (illustrated in Figure 1b). Assuming area-normalized rates, the proportionality constant is the squared energy-independent electronic coupling term between the electrode and the redox couple.^7,32^ Calculation of electronic coupling would require an electronic structure calculation of the ground-state atomic configuration, which is not feasible for the large moiré supercells characteristic of small-angle twisted multilayer graphene.

In low-dimensional electrodes with DoS variation,^37^ we account for the relatively small and potential-dependent quantum capacitance *C*_q_, which creates a dynamic doping effect,^31,38^ by shifting the DoS alignment by *eV*_q_, as indicated in eq 2. In bilayer and trilayer graphene, the dielectric capacitance is high enough that this effect can be neglected,^39^ as the voltage mainly drops across the quantum and the electrical double layer (EDL) capacitances. A schematic in the SI (Figure S6) shows the equivalent circuit and Fermi level adjustments after voltage drop. Assuming the rigid-band approximation, these quantities are obtained from the following coupled system of equations^17,40,41^
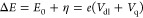
3a
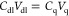
3b
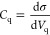
3cwhere Δ*E* is the difference in the Fermi levels of the redox couple and the electrode (Figure S6c) and is equal to the formal energy difference (*E*_0_) at equilibrium (Figure 1b). *V*_q_ and *V*_dl_ are voltage drops across quantum (*C*_q_) and EDL capacitances (*C*_dl_) respectively (Figure S6a). Capacitances in series hold the same charge and satisfy the requirements of eq 3b. *C*_q_ is given by the differential of excess charge density (σ) with voltage. Assuming that the electrochemical potential can shift rigidly with *eV*_q_ (Figure S6d), the excess charge density is given by^40,41^
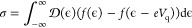
4

Substituting eq 4 in 3c gives a simplified expression for *C*_q_
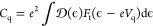
5a
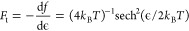
5bwhere *F*_t_ is the energy differential of the Fermi function, also called the thermal broadening function. Hence, the electrochemical potentials at the interface equilibrate to the same energy (Figure S6(d)) due to the DoS being offset by *eV*_dl_ and band filling by *eV*_q_. Previous calculations on tBLG^17^ suggest that a greater fraction of *E*_0_ goes into *V*_q_ near the CNP from flat electronic bands. This contributes to a small offset in the rate enhancement away from the MA, also observed in tBLG.^17^

The product of the first two factors (redox couple states and Fermi function) in the integrand of eq 2 almost approximates a Gaussian filter being convolved with the DoS. As illustrated in Figure 1c, when this filter is closer to the flat-band energy, the reaction rate is higher due to a greater overlap with the DoS. A number of parameters can be tuned that change the shape and position of the overlapping functions in the integrand. Figure 1b highlights the tunable parameters of the solvent (*E*_0_, λ) and the electrode (θ_12_, θ_23_, η). The temperature (*T*) broadens both the redox couple states and the Fermi function.

Using this model, we have shown a qualitative agreement with experiments in tBLG, where the rate enhancement is observed near the MA (1.1°).^17^ Notably, the observed enhancement is ∼10× higher than the theoretical prediction. The quantitative mismatch occurs primarily at the AA domains (Figure 5a by Yu et al.^17^), suggesting a substantial variation in the electron-coupling constant with spatial coordinates. Evaluation of this dependence on the space and twist angle is beyond the scope of this work. Modifications to the coupling constant like those studied on graphene edges^42,43^ and metallic electrodes^44,45^ can help narrow the gap between theory and experiment.

In the trilayer system, we employ a low-energy momentum-space continuum model, or the Fourier transform of the real-space tight-binding model, for DoS calculations.^18^ The model includes out-of-plane relaxation.^46,47^ Additionally, when the twist angles are both ≥1^°^, the in-plane relaxation and its effect on the DoS is small.^46,48^ The model utilizes one twist angle between each adjacent pair of layers (θ_12_ and θ_23_, measured in opposite senses but both defined to be > 0, see Figure 1a). The main advantage of the continuum model is that it can be used for arbitrary angles and any large-sized systems. It is not possible to do density functional theory (DFT) calculations for the small twist angles because of the lack of periodicity and large system size. For reference, the number of atoms scales as 1/θ^4^ which amounts to 65 million atoms at ∼1° twists. Even with the state-of-the-art DFT calculations, the reported limit is 600,000 electrons.^49^ In tBLG, the tight-binding Hamiltonian has been shown to match with the ab initio calculated band structure^50^ (Figure S8) and with observed insulating states.^51^ In the trilayer system, the zero-energy flat bands from the code agrees with the observed semimetallic resistance peaks^52^ and closely aligns with recent experimental findings.^53−55^ As a result, we have a high level of confidence in the accuracy of the DoS. An expression to quantify the uncertainty in rates from DoS has been derived in the Supporting Information. Maximum relative uncertainty in rates is found to be ∼1.8% based on Figure S8. To achieve a higher resolution in the DoS, we increased the momentum cutoff radius and the number of bands.

## Results and Discussion

**Figure 2:**
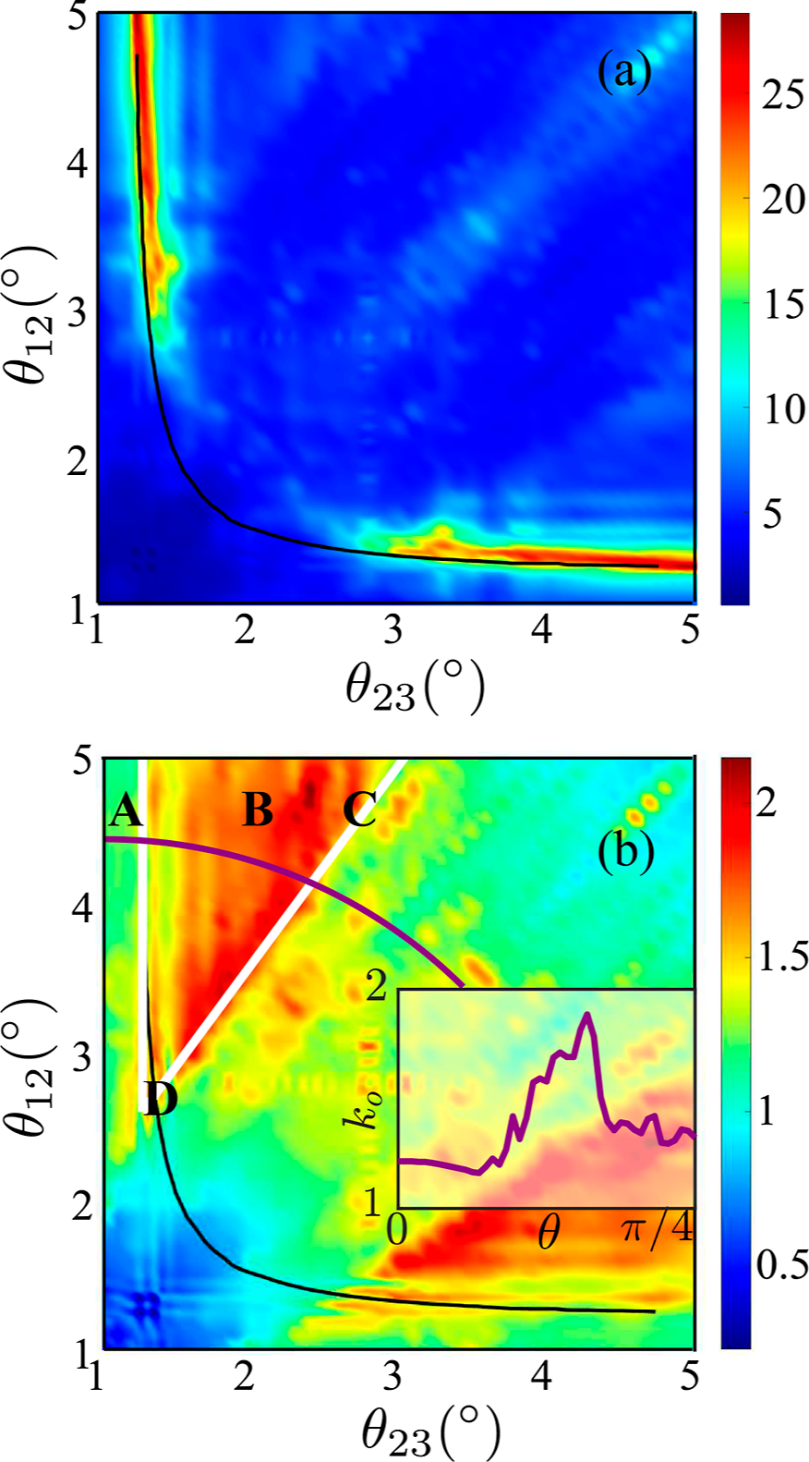
(a) Maximum DoS peak (Reproduced with permission from Zhu et al.,^18^ Copyright 2020 by the American Physical Society). (b) *k*_0_ rate map for RuHex (*E*_0_ = −0.07 eV, λ = 0.82 eV, η = 0 eV) as a function of the two independent twist angles in the tTLG system. The “magic angle curve” is shown by the black line. A number of weak hotspots in *k*_0_ (b) occur near the diagonal due to numerical artifacts in the DoS. The solid white triangle encloses the activity volcano, also marked by **A**, **B**, **C**, and **D**. Inset shows variation of *k*_0_ across a 45° arc as indicated. All rates are relative to the Bernal stacked (untwisted) ABA system. Maximum rate enhancement in the volcano is 2.1× over ABA.

The degree of twist modulates the van Hove singularity (VHS) separation and associated DoS peaks (Figure 1b) arising from the flat bands. Figure 2a shows the magnitude of these peaks at a range of twist angles (reproduced from Zhu et al.^18^). The high-magnitude peaks asymptotically converge to tBLG MA (1.1°) when the other layer is decoupled (twisted at a large angle >4^°^). Plugging the model DoS into the MHC model, we see that the reaction rate contour (*k*_0_) for RuHex (Figure 2b) contrasts starkly with the DoS peak contour. The color magnitudes are shown relative to the reference ABA (Bernal stacked) system for direct visualization of the rate enhancement. The comparison assumes no change in electronic coupling with the redox couple between different twist angles. Unlike tBLG, the rate enhancement is not confined to the MA curve but spans a sizable triangular area (volcano), as indicated in Figure 2b.

**Figure 3:**
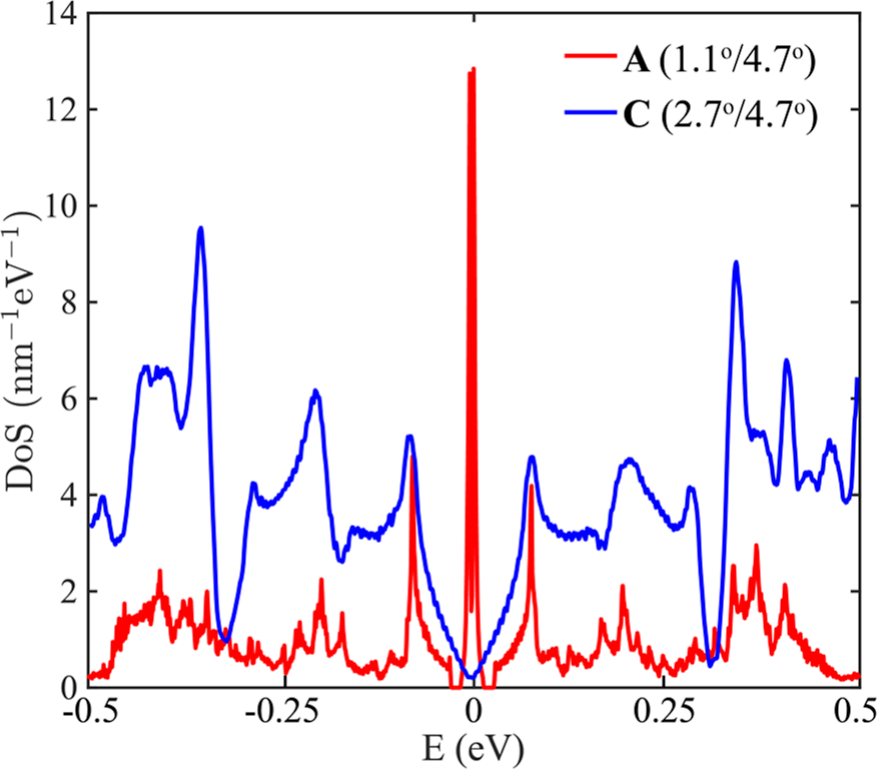
Density of states of tTLG at the magic angle **A** (1.1°/4.7°, red) and at the incommensurate angle **C** (2.7°/4.7°, blue).

In this region, the value of *k*_0_ is ∼2.1× higher than untwisted ABA, which is similar to the theoretical enhancement reported for tBLG (2.2 ×)^17^ with respect to the Bernal stacked AB graphene. Magnitude-wise, *k*_0_ in the tTLG volcano is 3.2× higher than tBLG at 1.1°, which is expected from the flatter DoS of the former (comparing flat bands in Figure 3 and S4a). Experimentally, this could translate to more than an order of magnitude enhancement in tTLG compared to that in tBLG. We also show the volcano peak across a 45° arc passing over the enhancement region (Figure 2b inset).

The activity volcano starts at the MA curve (line **AD**, Figure 2b) where the DoS peaks are the sharpest. Going horizontally from **A** to **C**, the VHS separates toward higher energies (Figure 3), similar to tBLG. In tTLG, however, there is an additional change in the band hybridization from commensurate to incommensurate angles, as shown by Zhu et al.^18^ in Figure 1b. These changes are frequent from **A** to **B**, where **B** is at the dominant (2,1) moiré harmonic line (Figure 1b^18^). After **B**, there is a significant region of incommensurate angles until the (1,1) harmonic, in which many competing harmonics coexist, and nonoverlapping bands occur over different energy ranges. These bands show an increased DoS in the energy range ±0.25 eV (Figure 3), where the DoS has an optimal overlap with the MHC filter (Figure S1). Consequently, the DoS area within ±0.25 eV increases from **B** to **C** (Figure S5), producing a high value for *k*_0_ in this region. After **C**, the incommensurate states fall outside the filter range, and *k*_0_ diminishes quickly. These nonoverlapping bands are not found in polytypes of tTLG with a single twist angle (Figure S4) like “monolayer-twist-bilayer” (M*t*B) and alternating twist (A*t*A) and are expected to enhance rates only near the MA.^35^ For visualization, a schematic of the local DoS of tTLG at points **A** and **C** over 100 nm is depicted in Figure S7. A function analogous to the generalized stacking fault energy^48^ is used to mimic the local DoS.

The vertex of the volcano (**D**) starts at the point where the (2,1) harmonic line meets the MA curve.^18^ Therefore, Δ**ABD** is enhanced by the flat bands, and Δ**BCD** by nonoverlapping bands from incommensurate angles. Near standard conditions, the whole area is kinetically enhanced because both effects are equally significant. In our rate eq 2, temperature governs several key terms, such as the prefactor, the thermal broadening of Fermi occupations, and the electrode and redox couple states inside the integral. The finite temperature effect is embedded in our DoS calculations via Gaussian smearing. In theory, lower temperatures should promote flat bands by enabling better overlap with a tighter filter. Conversely, higher energy resonant states at incommensurate angles should exert a greater influence at elevated temperatures due to thermal broadening. Assuming that the reorganization energy varies weakly with temperature,^56^ our calculations suggest that these effects only become prominent within a temperature range where solvent parameters change drastically from phase transitions. In addition, we lack experimental support on the temperature dependence of kinetic rates in tBLG. Due to these limitations, we only make qualitative assessments of the expected trend.

Other redox couple parameters (*E*_0_ and λ) can be adjusted to study the sensitivity and possible rate improvement. As shown in Figure S2, a higher λ reduces the overlap of the redox couple states with the Fermi electron/hole occupations, resulting in a decrease in the filter peak. Unlike the temperature, however, there is no effect on the filter width. Hence, while the rate magnitude varies, the relative enhancement area is unchanged with perturbations in λ (Figure S3). These observations suggest the possibility of engineering pairs of redox couples and solvents with low λ values for high values of *k*_0_.

**Figure 4:**
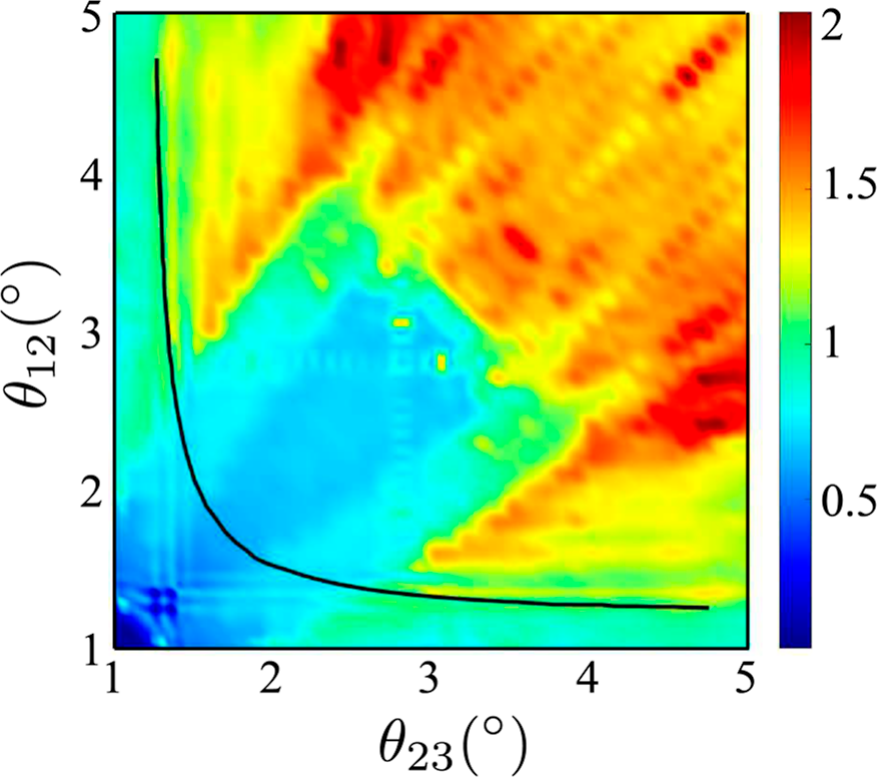
Equilibrium rate constant map at *E*_0_ = 0.3 eV; *T* = 300 K and λ = 0.82 eV. The solid black line marks the magic angle curve for tTLG. The color scheme indicates rates relative to the untwisted ABA system. The enhancement area (red) imitates the incommensurate angle region (purple) reported by Zhu et al.^18^ Activity is compromised at the magic angle due to the high chosen formal potential of the redox couple.

One can also employ redox couples with varying formal potentials (*E*_0_) to enhance different twist angle regimes in the trilayer. Higher values of *E*_0_ would compromise activity at the magic angle by diminishing the interaction with the flat bands, favoring resonant states at higher energies from incommensurate angles. This is evidenced by Figure 4, where at *E*_0_ = 0.3 eV, we observe a shift in the rate enhancement to higher incommensurate angles corresponding to the purple region of the dominant moiré harmonics plot (Figure 1b^18^). Hence, previously inactive redox couples in tBLG like cobalt phenanthroline (CoPhen) and ferrocene methanol (FcMeOH)^17^ (*E*_0_ ∼ 0.3 eV) may achieve comparable activity with RuHex at the incommensurate angles. To boost the effect from magic angles, low-*E*_0_ couples (such as RuHex) energetically align with the flat bands.

## Conclusions

In conclusion, we have explored the role of incommensurability in identifying regions of unexpected rate enhancement in tTLG, which was otherwise “locked” at the magic angles in commensurate systems like tBLG, M*t*B, and A*t*A systems. Incommensurability introduces nonoverlapping bands that increase the number of interacting states at higher energies, thus magnifying equilibrium rates away from the MA curve. We also identified a new perspective on the factors involved in the MHC formalism and its role in the control of parameters. For instance, temperature and redox couple formal potentials can adjust rate magnitudes and switch enhancement between MA and the incommensurate angles. Further study is required to verify the temperature effects, as support from experiments in tBLG is lacking. In standard conditions, we predict the emergence of a high-activity volcano spanning both MA and incommensurate regions using the RuHex redox couple. The rate enhancement in experiments is expected to be greater as observed with tBLG. Moreover, redox couples with greater values of formal potentials such as CoPhen and FcMeOH can activate higher incommensurate angles at the expense of MA.

A limitation of the present study is that we have not accounted for the twist dependence of the electron coupling between the electrode and the redox couple. Its role and magnitude on MA and incommensurate angles will be explored in future studies. This work presumes an outer-sphere redox reaction and hence a weak adsorption energy of the redox couple. We consider this assumption valid since the physisorbed energy of molecules on small-angle tBLG is ∼26 meV weaker compared to monolayer and other large-angle tBLG.^57,58^ This trend is attributed to stronger interlayer coupling at small-angle twists, leading to weaker adsorption. Further investigation using ab initio energy calculations^58,59^ and cyclic voltammetry^60,61^ can be performed to determine the spatial variation of adsorption energy near magic and incommensurate angles. While we focus here on trilayer, incommensurability occurs more frequently in multilayer 2D systems, indicating immense flexibility in maintaining high activity for a wide range of electrochemical processes. Moreover, localization of charge density between layers will likely emerge as an additional tuning parameter for optimization, as demonstrated recently.^35^ Our work thus opens a new field of kinetics in 2D materials, capturing the signatures of the commensurate and incommensurate moiré structures and finding an optimal balance of charge-transfer reactivity.

## Data Availability

The data and code that support the findings of this study are openly available on Github.^62^

## Supporting Information Available

Evolution of the filter shape (product of Marcus and Fermi functions) with temperature and reorganization energy, equilibrium rate map at λ = 0.2, 1.2 eV, DoS of bilayer, M*t*B and A*t*A graphene at two twist angles, map of trilayer integrated DoS between ±0.25 eV as a function of twist angles, schematic of quantum and EDL capacitances at the interface of electron transfer, schematic of local DoS for commensurate and incommensurate angle tTLG, and a section that quantifies uncertainty in rates from DoS (PDF)
