# Supplementary material for: Twisto-Electrochemical Activity Volcanoes in Trilayer Graphene
Source: J Am Chem Soc. 2024 Jun 3;146(23):16105–11. doi: 10.1021/jacs.4c03464 (PMC11177310; doi:10.1021/jacs.4c03464)
Supplement: Supplementary file 1 — ja4c03464_si_001.pdf [file ja4c03464_si_001.pdf]

Supporting Information: Twisto-Electrochemical Activity Volcanoes in Trilayer Graphene

Mohammad Babar,[1] Ziyan Zhu,[2,3] Rachel Kurchin,[4]
Efthimios Kaxiras,[3] and Venkatasubramanian Viswanathan[5]

[1]*Mechanical Engineering Department,*
University of Michigan, Ann Arbor, Michigan 48105, USA
[2]*Stanford Institute of Materials and Energy Science,*
SLAC National Accelerator Laboratory, Menlo Park, CA 94025, USA
[3]*Department of Physics, Harvard University,*
Cambridge, Massachusetts 02138, USA
[4]*Department of Materials Science and Engineering,*
Carnegie Mellon University, Pittsburgh, Pennsylvania 15213, USA
[5]*Aerospace Engineering Department,*
*University of Michigan, Ann Arbor, Michigan 48105, USA**

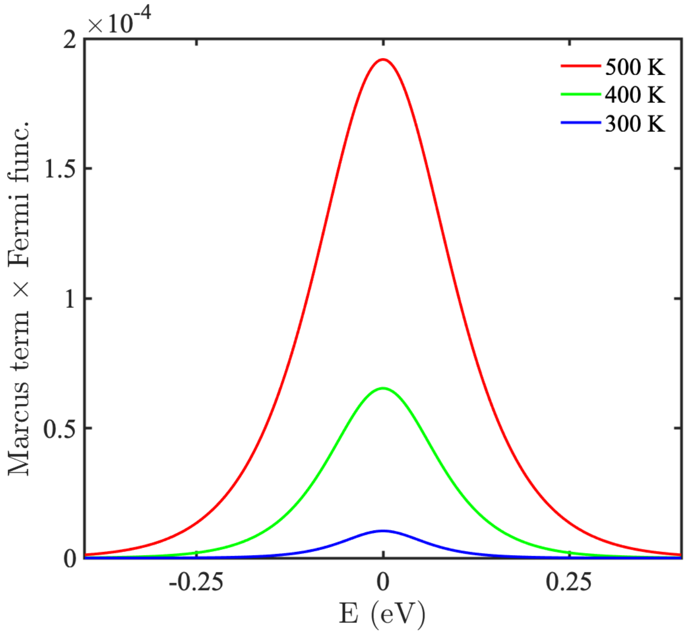

FIG. S1. The product of Marcus and the Fermi functions at different temperatures, which acts as an energy filter of the density of states.

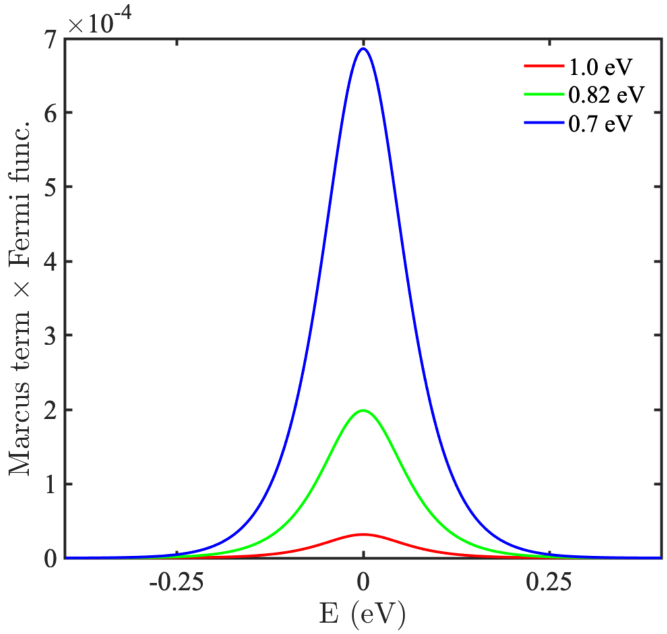

FIG. S2. The product of Marcus and the Fermi functions at different reorganization energies.

The product of the Marcus and Fermi functions inside the rate integrand is shown at different temperatures (fig. SS1) and reorganization energies (fig. SS2). This product overlaps with the density of states to give the overall rate integrand, and is referred to as the MHC

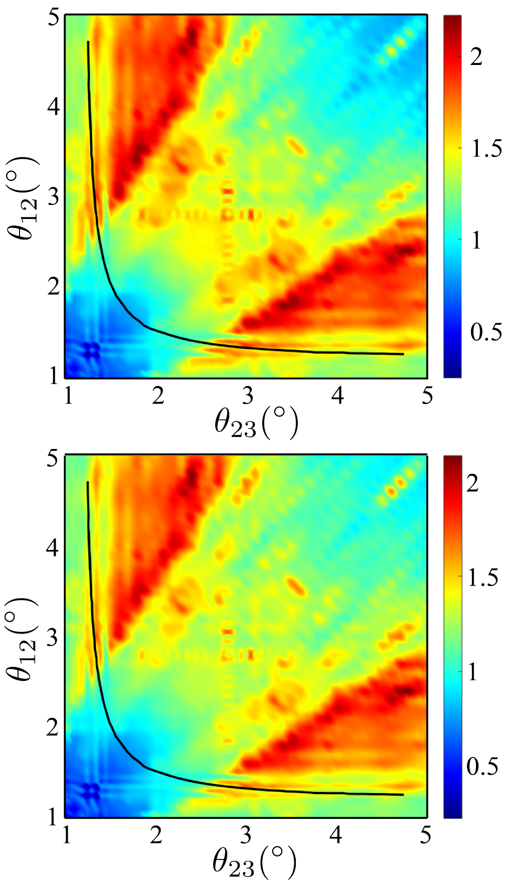

FIG. S3. Equilibrium rate maps for (a) λ = 0.2 eV and (b) 1.2 eV for RuHex solvent. The rate enhancement region is almost unaffected since the integrand has a constant energy spread with λ.

filter in the main text. The filter peak and width increase with temperature. The optimal energy range for high overlap of the density of states with the MHC filter is $\pm$0.25eV. Increasing the reorganization energy reduces the peak but the energy spread of the product is not affected. The effect of this is exemplified by the almost identical equilibrium rate maps for two reorganization energies (λ = 0.2eV, 1.2eV) at standard values for RuHex (T=295 K, E_0=-0.07 V, η=0) as shown in fig. SS3.

The DoS peaks are flatter in tTLG than in tBLG (fig. 3 main text vs fig. SS4(a)), the equilibrium rate therefore is higher ($\times$2) in the former system at its MA. Like tBLG, polytypes of tTLG, MtB and AtA are commensurate at all twist angles, and do not show filled band gaps from non-overlapping states at higher energies ($\sim$ $\pm$0.25 eV) away from their MA (fig. 3 main text vs fig. SS4). As a result, maximum activity in commensurate systems is "locked" at the MA. Compared to tBLG, these polytypes of trilayer graphene

have flatter bands from correlated electronic phases (fig. SS4) and are predicted to show an order of magnitude higher value of k_0 in the experiments [1].

The map of integrated DoS in ± 0.25 eV (fig. SS5) indicates gradually increasing values of DoS area around point **C** (fig. 2b of the main text). The maxima occurs at $(2.7^\circ/-4.7^\circ)$. As explained in the main text, a combination of factors are responsible for this behaviour, (a) increase in VHS peak with twist angles from expanding Brillioun Zone (b) flips in band hybridization in the twist angle range. The second factor explains the increase in DoS area from $2.2^\circ/-4.7^\circ$ (commensurate) to $2.7^\circ/-4.7^\circ$ (incommensurate) angles. The DoS area drops after **C** as incommensurate states move out of the integration range (filter width ± 0.25 eV).

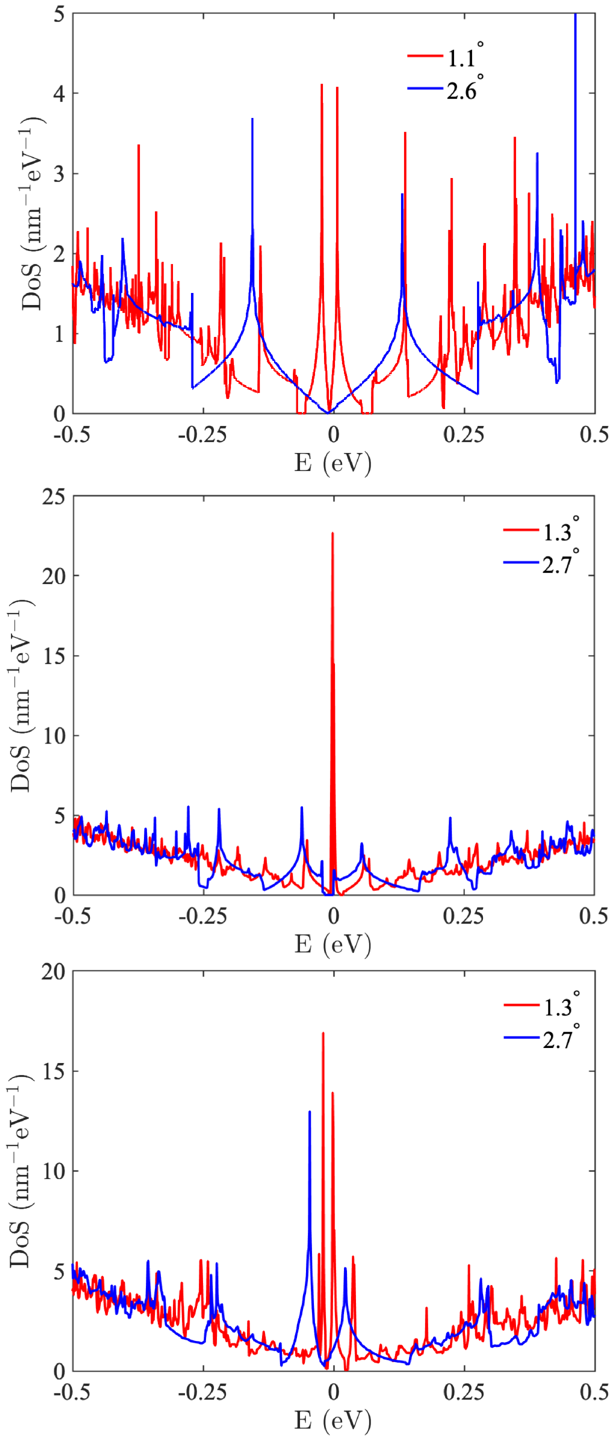

FIG. S4. Density of states of commensurate systems near (red) and away (blue) respective magic angles, (a) twisted bilayer graphene (tBLG), (b) monolayer twisted bilayer (MtB) and (c) alternating twist trilayer (AtA).

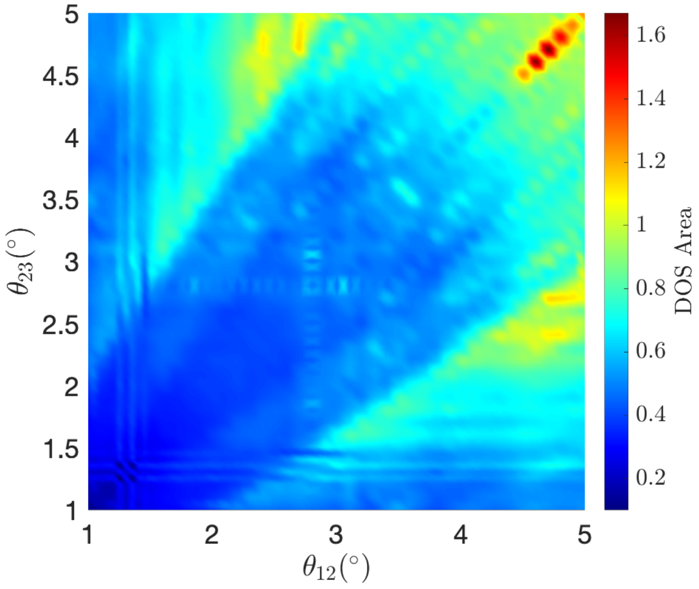

FIG. S5. Integrated tTLG DoS in ±0.25 eV as a function of twist angles (θ_{12} and θ_{23}). The DoS area is highest around **C** ($2.7^\circ/-4.7^\circ$) within the integration limits. Intense red spots near $\theta_{12}, \theta_{23} = 5^\circ$ are due to the numerical artifacts in the DoS.

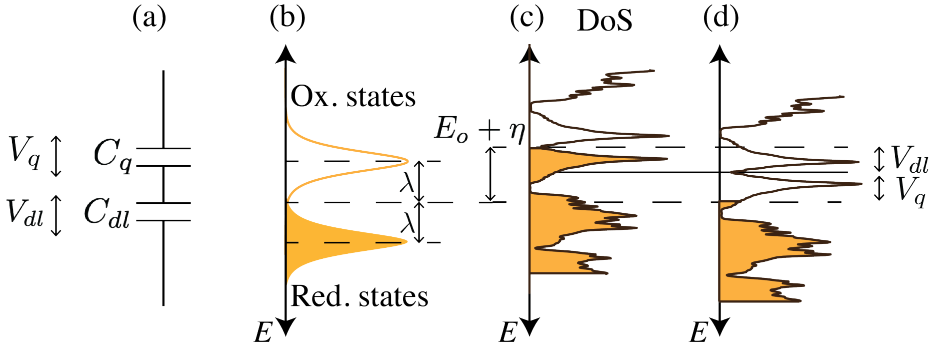

FIG. S6. Schematic of the series quantum and EDL capacitances at the interface of electron transfer. From the left (a) equivalent circuit, (b) redox couple oxidation and reduction states, DoS of the electrode before (c) and after (d) Fermi levels adjust at same energy.

(a)

(b)

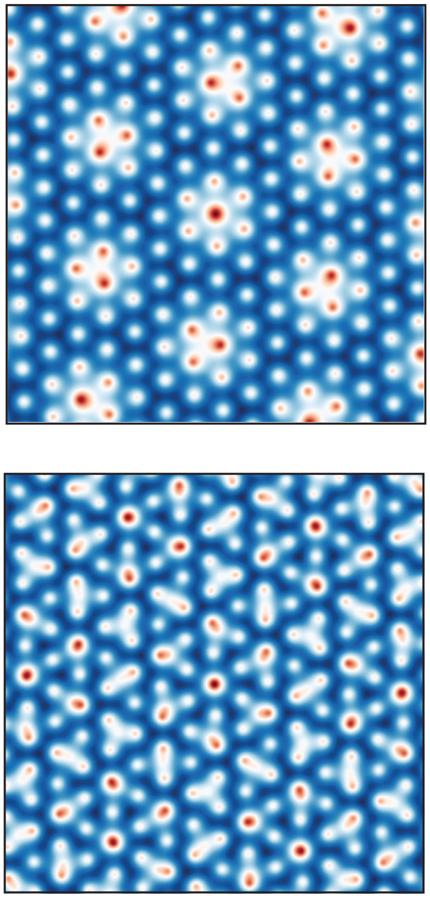

FIG. S7. Schematic of the tTLG system that mimics the local DoS for commensurate **A** $(1.1^\circ/-4.4^\circ$, a) and incommensurate **C** $(2.7^\circ/-4.7^\circ$, b) angles in the main text. The limit of x- and y-axes is 100 nm in real space. Commensurate magic angle forms a periodic lattice with sharply peaked DoS at AA spots between the bilayer. In contrast, the pattern is non-periodic and spatial domains are not distinguishable for the incommensurate angle.

UNCERTAINTY IN RATES FROM DOS

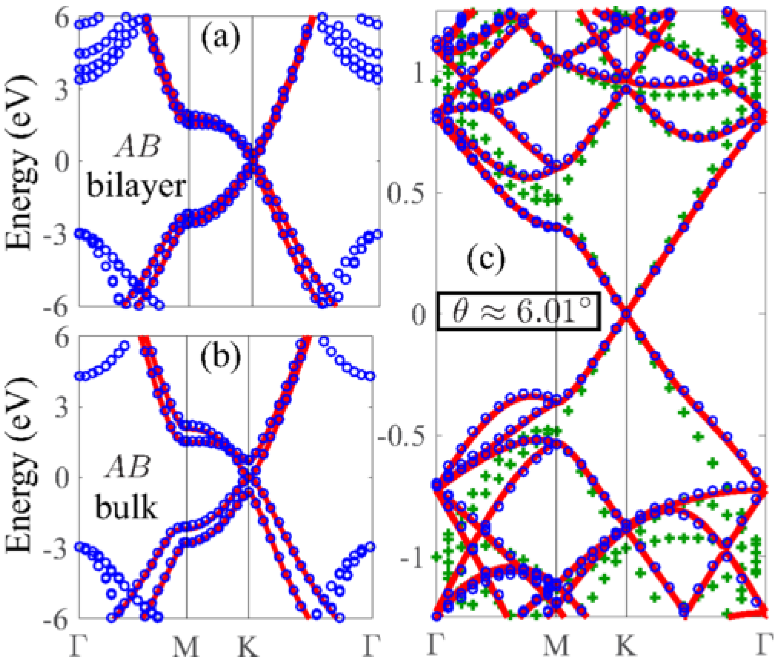

FIG. S8. Comparison between the tight-binding Hamiltonian (red lines) and ab initio DFT (blue circles) band structure calculations. Reproduced from Fang and Kaxiras [2]. Copyright 2016 by the American Physical Society.

For an integral equation,

$$I(a) = \int_{x_0}^{x_1} F(a)\mathrm{d}x \tag{1}$$

with uncertainty in a will lead to,

$$\delta I(a) = \frac{\partial I}{\partial a}\delta a. \tag{2}$$

Partial derivatives can be shifted inside the integral,

$$\frac{\partial I}{\partial a} = \int_{x_0}^{x_1} \frac{\partial F}{\partial a}\mathrm{d}x. \tag{3}$$

Based on these equations, the uncertainty in rates due to DoS in the MHC-DoS theory is,

$$\delta k = A\left(\frac{1}{\sqrt{4\pi\lambda k_{\mathrm{B}}T}}\int \exp\left(\frac{-(\epsilon+\eta+\lambda)^2}{4\lambda k_{\mathrm{B}}T}\right) f(\epsilon, T)\mathrm{d}\epsilon\right)\delta\mathcal{D} \tag{4}$$

where A is the prefactor. The integrand is the filter function defined in the main text (product of Marcus and Fermi functions) which at equilibrium is identical for oxidation and reduction. We skip uncertainty in the offset eV_{q} as it integrates DoS further into the

excess charge density (Eq.(4) in the main text). Using the average DoS value, the relative uncertainty is given by,

$$\frac{\delta k}{k} = \mathcal{D}_{avg} \left(\frac{\int \exp\left(\frac{-(\epsilon+\eta+\lambda)^2}{4\lambda k_{\mathrm{B}} T}\right) f(\epsilon, T) \mathrm{d}\epsilon}{\int \exp\left(\frac{-(\epsilon+\eta+\lambda)^2}{4\lambda k_{\mathrm{B}} T}\right) f(\epsilon, T) \mathcal{D}(\epsilon + eV_{\mathrm{q}}) \mathrm{d}\epsilon} \right) \frac{\delta \mathcal{D}}{\mathcal{D}_{avg}} \tag{5}$$

The maximum value of this expression is $0.9\times$ the relative uncertainty of DoS. As expected, the value is lower than unity since error on integration decreases. From fig. SS8, the average error in the tight-binding DoS is $\sim 2\%$, thus error propagated to kinetic rates is small $\sim 1.8\%$.

* venkvis@umich.edu

[1] K. Zhang, Y. Yu, S. Carr, M. Babar, Z. Zhu, B. J. Kim, C. Groschner, N. Khaloo, T. Taniguchi, K. Watanabe, *et al.*, ACS central science **9**, 1119 (2023).

[2] S. Fang and E. Kaxiras, Physical Review B **93**, 235153 (2016).
